# Comparison of the Effect of Toothbrushing Education Via Video, Lecture and Pamphlet on the Dental Plaque Index of 12-Year-Old Children

**DOI:** 10.3390/children5040050

**Published:** 2018-04-14

**Authors:** Javad Ramezaninia, Mohammad Mehdi Naghibi Sistani, Zohreh Ahangari, Hemmat Gholinia, Iman Jahanian, Samaneh Gharekhani

**Affiliations:** 1 Faculty of Dentistry, Babol University of Medical Sciences, Babol 47176-47745, Iran; javaderamezani91@yahoo.com; 2 Department of Community Oral Health, Faculty of Dentistry, Babol University of Medical Sciences, Babol 47167-47745, Iran; m.naghibi@mubabol.ac.ir; 3 Oral Health Research Center, Faculty of Dentistry, Babol University of Medical Sciences, Babol 47167-47745, Iran; s.ahangari69@gmail.com; 4 Health Research Institute, Babol University of Medical Sciences, Babol 47176-47745, Iran; h_gholinia@yahoo.com; 5 Department of Medical Education, Education Development Center, Babol University of Medical Sciences, Babol 47176-47745, Iran; i.jahanian@mubabol.ac.ir; 6 Oral Health Research Center, Faculty of Dentistry, Babol University of Medical Sciences, Babol 47167-47745, Iran

**Keywords:** adolescent, education, lecture, pamphlet, toothbrushing, video

## Abstract

The aim of this study was to compare the effect of different modes of toothbrushing education (lecture, video and pamphlet) on the dental plaque index (PI) of adolescents. The cluster randomized intervention was performed on 128 participants aged 12 years, who were allocated into four groups based on the type of intervention. Group 1: no intervention; and groups 2, 3, 4: education via lecture, video, and pamphlet, respectively (*n* = 32). Their plaque index was measured at the baseline, 24 h and two months later. Data were analyzed by repeated measures analysis of variance (ANOVA), one-way ANOVA, independent and paired *t*-test. The plaque indices of groups 2, 3, 4 at 24 h (*p* values < 0.001) and two months (*p* values < 0.001) showed a significant reduction when compared to the baseline. The lowest PI score was observed in the pamphlet, video and lecture groups at 24 h, respectively. After 2 months, the lowest score of PI was measured in lecture, video and pamphlet groups, respectively; however, these differences were non-significant. Therefore, toothbrushing education via lecture, video and pamphlet reduced the dental plaque index with the same effectiveness.

## 1. Introduction

Poor oral hygiene is related to microbial plaque aggregation on the teeth and oral structure which results in dental caries [1], gingivitis and periodontal disease [2]. Several studies have reported a high prevalence of caries and periodontal disease in Iranian children and adolescents [3,4,5,6,7,8,9]. Oral hygiene education has been shown to be effective, specifically among 9–12-year-olds who are responsible for their oral hygiene themselves, and parents now become the observer instead of their previous active role. In addition to lower cooperation and motivation for health among 12-year-old children, reduced self-confidence at the same time as puberty leads to poor plaque control [9,10]. Changes in diet and hormones can also cause caries development and inflammation. As a result, it is becoming increasingly important to retrain and motivate children at this age [10].

The three major methods for oral health education comprise of verbal, written and audiovisual methods [9].

Jabarifar et al. concluded that the lecture method was more effective than video for the promotion of oral health knowledge, attitude and practice among 14-year-old children [11]. However, controversies exist regarding the most effective oral health education methods. Redmond et al. reported a higher acceptance rate of written health education methods among adolescents [12], while educational videos resulted in a greater effect on oral hygiene learning when compared to the lecture method [13]. Therefore, the aim of this study was to compare the effect of different educational methods of toothbrushing (lecture, video and pamphlet) on the dental plaque index (PI) of 12-year-old children.

## 2. Materials and Methods

### 2.1. Participants

The cluster randomized interventional trial was performed from January to April 2017 on 12-year-old children in public and private schools in Babol, north of Iran. A sample size of 128 participants was considered (*α* = 0.05, power = 80%, effect size = 0.6). The subjects were allocated into four groups based on the type of intervention: group 1 had no intervention; group 2 had a lecture; group 3 watched a video; group 4 read a pamphlet (*n* = 32), and they were matched according to gender and school type. In each school, just one educational group was selected.

The inclusion criteria consisted of healthy children, having teeth take part in the plaque index (11, 16, 26, 31, 36 and 46). The exclusion criteria included the dissatisfaction of children to participate in the study, physical and mental problems that limited learning ability, having a painful tooth in the mouth, sensitivity to toothpaste, and having a large dental calculus.

### 2.2. Clinical Measurements and Outcomes

All study groups (including the control) received a toothbrush/toothpaste before the baseline PI assessment and had an opportunity to brush before the assessment. The following program was performed for the intervention groups. At the baseline, after measuring the plaque indices, the participants were trained in the Bass toothbrushing technique [14]. Next, the plaque indices were assessed 24 h later. The retraining was delivered four weeks later for all groups and was the same as teaching at the baseline. The final plaque index measurement was done one month later (eight weeks after the baseline).

**Figure 1:**
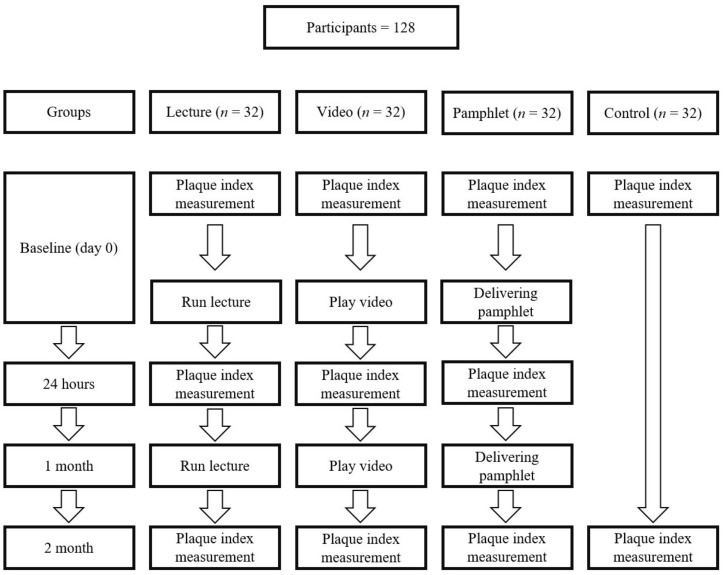
Study procedure.

In addition, the plaque index assessment served for no intervention group (group 1) at the baseline and two months later. Figure 1 displays the study procedure.

The plaque index measurement was performed for all 128 participants by two well-trained, but not blinded senior dental students (intra- and inter-examiner v *κ* value = 0.91 and 0.86) during school hours at the health office of the school on a comfortable chair, using a mouth mirror and a dental explorer.

A single toothbrush and toothpaste (Oral-B, Iowa City, IA, USA) were given to each student and they were asked to brush their teeth, then the Sillness and Loe plaque indices were measured [15].

The mean PI scores were classified as less than 0.1 (absence of plaque or perfect plaque control), between 0.1 to 1.0 (small amount of plaque or good plaque control), between 1.0 to 2.0 (average value or fair plaque control), and between 2.1 to 3 (bad plaque control).

### 2.3. Educational Intervention

The content of the video, pamphlet and lecture was collected from a text of pediatric dentistry and Carranza’s clinical periodontology [10,15], and included a brief introduction on dental plaque and tooth decay development and an explanation of the Bass toothbrushing technique, which was evaluated by two pedodontists from the Babol Faculty of Dentistry. The educational content of the video and pamphlet was assessed by one of the masters in the Education Development Center (EDC) department of the Babol University of Medical Sciences.

The video was made in two sections including an animation describing dental plaque and caries development and a film with a footnote in the Persian language displaying the toothbrushing method on a dental model for eight minutes.

The lecture was also presented by a senior dental student within almost 10 min. The lecture was accompanied by brushing training on a dental model. The pamphlet was designed with both text and images in the Persian language and was given to participants for studying within 10 min. The content of the intervention was the same across the three modes of delivery.

All interventions were collectively conducted by a senior dental student in each group. The participants were allowed to ask questions about what was unclear during their education and they were questioned randomly in order to gain active participation.

### 2.4. Statistical Analysis

The differences in plaque reduction were evaluated using repeated measures analysis of variance (ANOVA), one-way ANOVA, and the independent *t*-test and paired *t*-test. The significance was set at 0.05.

### 2.5. Ethical Considerations

This study was approved by the Ethics Committee (No. MUBABOL.REC.1395.260, date 2017/03/12) and Oral Health Research Center of the Babol University of Medical Sciences (Babol, Iran). Parents were given informed consent. The study protocol was submitted to the Iranian Registry of Clinical Trials (IRCT 2017080721519N5).

## 3. Results

In this interventional study no participants were missed. The PI mean (± standard deviation (SD)) of 12-year-old children at the baseline was 0.89 (±0.41). A good status of oral and dental hygiene was considered when 0.1 ≤ PI ≤ 1. A mean score of PI at the baseline according to the interaction groups (control, lecture, video and pamphlet), gender, and type of school are represented in Table 1. The PI mean at the baseline in public schools was significantly lower than that of private schools (*p* = 0.002). Among the study groups, the PI mean in the pamphlet group was significantly higher than that of the control group (*p* = 0.01).

Table 2 displays the mean PI for the intervention groups at different time periods. In the control group, a mean PI score at the two-month interval increased significantly when compared to the baseline (*p* = 0.01), while in all educational groups a significant reduction occurred between 24 h and two months when compared to the baseline (*p* < 0.001).

The boys’ mean PI were lower than that of girls in the pamphlet group at two months (*p* = 0.03); however, no significant difference existed in all intervention groups by different times (baseline, 24 h and two months) in the mean PI score between girls and boys (Table 3).

## 4. Discussion

The main aim of this study was to compare the effect of toothbrushing education by practical lecture, video and a pamphlet on the dental plaque index among 12-year-old children. Like previous studies, the results indicated that education was effective in plaque reduction [9,16,17,18]. In this study, a statistically significant decrease occurred in the PI of all educational groups in both intervals after training (24 h and two months) when compared to the control group. Since the oral hygiene status of the subjects was estimated as “good” at the baseline, no significant clinical difference was seen after education. The control group showed a significant increase in the PI during the two months, which may be due to no participation in any educational or motivational intervention either in the framework of this research project or otherwise.

Among the study groups, the lowest score of PI was in the pamphlet, video and lecture at 24 h, respectively. This difference indicated the higher impact of the pamphlet in a short time. After two months, the lowest score of PI was observed in the lecture, video and pamphlet groups, respectively; however, these differences were non-significant.

A significant reduction in plaque index was observed after 24 h and two months when compared to the baseline which was similar in all study groups. In order to study the short-term effect of education, the plaque index was measured 24 h later; and for the long-term effect, it was evaluated eight weeks later. In order to enhance the degree of learning, the retraining program was planned four weeks after the baseline.

When comparing different oral health education methods, however, Yazdani and colleagues reported that leaflets were more effective than videotapes, while a culturally appropriate video revealed oral hygiene improvement among Nigerian children [9].

In the current study, although no significant PI difference was observed by gender in the baseline, boys in the pamphlet group showed more PI reduction than did the girls, while previous study in Tehran revealed that the videotape was more effective to improve boys’ oral cleanliness and gingival health when compared to girls [9]. This difference consists of different gender preferences in educational material in different geographical states in Iran, which may be related to socio-cultural factors. Therefore, oral health education interventions should consider gender preferences in different states in Iran.

The selection of private and public schools was considered according to their predictive role in the household economic situation. In the present study, students in private schools showed a higher PI when compared to public schools’ students at the baseline. Participants from both public and private schools, however, showed plaque reduction in all intervention groups.

Regarding learning styles, three main ways exist such as writing, audio-visual and oral methods [9]. In this study, the authors used the three main methods of health education including a pamphlet, lecture and video. The pamphlet served as the writing method regarding its advantages such as summarizing content, fast replication, simple design, reusability, and multi-threading ability. However, the pamphlet lacked the ability to transmit the message and the details of a practical tutorial completely.

The video served as the audio-visual method regarding its several advantages such as attractiveness and the possibility of repetition of similar training without modifying the quality and quantity of data for the group, as well as being easy to apply. However, this method needed an audio-visual player, which was inappropriate or defective at some schools.

Verbal education with a dental model needed less specialized equipment and facilities while allowing children to closely see how to brush.

Some limitations of this study included the lack of knowledge assessment of participants to ensure that the education content was completely paid attention to or understood, the fact that the examiner was not blind to the study groups, and that the authors could not possibly prevent the students from receiving additional educational information from other sources. Loyalty to the speaker’s text may not be the same across the different groups, and this was subject to limitation. While health education has benefits, the long-term effectiveness of oral health education interventions for improving oral health outcomes is questionable [19]. Asimakopoulou and Newton believed that behavioral changes resulted from capability, opportunity and motivation while knowledge was only part of capability [20].

Overall, in the present study, the effect of different educational methods on the PI was not significantly different.

## 5. Conclusions

Toothbrushing education with lecture, video and pamphlet modes reduced the dental plaque index. In all three different teaching methods, the effectiveness was almost the same; however, the pamphlet was the most effective method in a 24 h period. To achieve effective results, in the future, oral health promotion programs could use each of these oral health education methods set to the unique resources of the settings.

## Figures and Tables

**Table 1:** The mean (± standard deviation (SD)) plaque index (PI) in baseline by gender, type of school and interaction groups.

		Mean ± SD	*p*-Value
Gender	Girl	0.9 ± 0.43	0.9 *
	Boy	0.89 ± 0.38	
Type of school	Public school	0.78 ± 0.38	0.002 *
	Private school	1.00 ± 0.40	
Educational groups	Control	0.70 ± 0.40 (A)	0.01 **
	Lecture	0.89 ± 0.40 (AB)	
	Video	0.95 ± 0.38 (AB)	
	Pamphlet	1.02 ± 0.39 (B)	

There is no statistically significant difference between two mean plaque indices with same letters at 0.05; *p*-value *: independent *t*-test; *p*-value **: analysis of variance (ANOVA).

**Table 2:** Comparison of PI in interaction groups at different times.

Groups	Base	24 h	Two Months	*p*-Value
Control	(a) 0.70 ± 0.40	-	(a) 1.04 ± 0.40	0.01 ***
Lecture	(ab) 0.89 ± 0.40 (A)	(a) 0.30 ± 0.19 (B)	(b) 0.14 ± 0.12 (B)	<0.001 *
Video	(ab) 0.95 ± 0.38 (A)	(ab) 0.25 ± 0.15 (B)	(b) 0.18 ± 0.11 (B)	<0.001 *
Pamphlet	(b) 1.02 ± 0.39 (A)	(b) 0.19 ± 0.14 (B)	(b) 0.24 ± 0.20 (B)	<0.001 *
*p*-Value	0.01 **	0.04 **	<0.001 **	-

There is no statistically significant difference between two mean plaque indices with same letters at 0.05 (abc system for column comparison and ABC system for row comparison). *p*-Value *: repeated measures ANOVA; *p*-value **: one-way ANOVA; *p*-value ***: pair *t*-test.

**Table 3:** Comparison of PI in different groups by gender.

Groups	Gender	Base	24 h	Two Months	*p*-Value
**Control**	Girls	0.71 ± 0.40	-	0.98 ± 0.33	<0.001 ***
	Boys	0.70 ± 0.41	-	1.01 ± 0.46	<0.001 ***
	*p*-Value	0.95 **	-	0.40 **	-
**Lecture**	Girls	0.89 ± 0.33 (A)	0.30 ± 0.16 (B)	0.15 ± 0.13 (C)	<0.001 *
	Boys	0.89 ± 0.36 (A)	0.29 ± 0.22 (B)	0.13 ± 0.12 (C)	<0.001 *
	*p*-Value	1.00 **	0.60 **	0.90 **	-
**Video**	Girls	1.05 ± 0.42 (A)	0.25 ± 0.18 (B)	0.16 ± 0.08 (B)	<0.001 *
	Boys	0.86 ± 0.32 (A)	0.24 ± 0.12 (B)	0.20 ± 0.13 (B)	<0.001 *
	*p*-Value	0.16 **	0.28 **	0.83 **	-
**Pamphlet**	Girls	0.94 ± 0.42 (A)	0.14 ± 0.09 (B)	0.27 ± 0.27 (B)	<0.001 *
	Boys	1.10 ± 0.35 (A)	0.24 ± 0.16 (B)	0.21 ± 0.11 (B)	<0.001 *
	*p*-Value	0.24 **	0.47 **	0.03 **	-

There is no statistically significant difference between two mean plaque indices with same letters at 0.05. *p*-Value *: repeated measures ANOVA; p-value **: independent *t*-test; *p*-value ***: pair *t*-test.
